# Differences in Counting Skills Between Chinese and German Children Are Accompanied by Differences in Processing of Approximate Numerical Magnitude Information

**DOI:** 10.3389/fpsyg.2018.02656

**Published:** 2019-01-08

**Authors:** Jan Lonnemann, Su Li, Pei Zhao, Janosch Linkersdörfer, Sven Lindberg, Marcus Hasselhorn, Song Yan

**Affiliations:** ^1^ Empirical Childhood Research, University of Potsdam, Potsdam, Germany; ^2^ Department of Education and Human Development, Leibniz Institute for Research and Information in Education (DIPF), Frankfurt am Main, Germany; ^3^ Center for Individual Development and Adaptive Education of Children at Risk (IDeA), Frankfurt am Main, Germany; ^4^ Institute for Psychology, Chinese Academy of Sciences, Beijing, China; ^5^ Department of Psychology, University of Chinese Academy of Sciences (UCAS), Beijing, China; ^6^ Faculty of Education, Beijing City University, Beijing, China; ^7^ Faculty of Arts and Humanities, University of Paderborn, Paderborn, Germany; ^8^ Department of Educational Psychology, Goethe-Universität Frankfurt am Main, Frankfurt am Main, Germany; ^9^ Department of Psychology and Methods, Jacobs University Bremen, Bremen, Germany

**Keywords:** approximate number system, subitizing, counting, cross-national comparison, preschool

## Abstract

Human beings are supposed to possess an approximate number system (ANS) dedicated to extracting and representing approximate numerical magnitude information as well as an object tracking system (OTS) for the rapid and accurate enumeration of small sets. It is assumed that the OTS and the ANS independently contribute to the acquisition of more elaborate numerical concepts. Chinese children have been shown to exhibit more elaborate numerical concepts than their non-Chinese peers, but it is still an open question whether similar cross-national differences exist with regard to the underlying systems, namely the ANS and the OTS. In the present study, we investigated this question by comparing Chinese and German preschool children with regard to their performance in a non-symbolic numerical magnitude comparison task (assessing the ANS) and in an enumeration task (assessing the OTS). In addition, we compared children’s counting skills. To ensure that possible between-group differences could not be explained by differences in more general performance factors, we also assessed children’s reasoning ability and processing speed. Chinese children showed a better counting performance and a more accurate performance in the non-symbolic numerical magnitude comparison task. These differences in performance could not be ascribed to differences in reasoning abilities and processing speed. In contrast, Chinese and German children did not differ significantly in the enumeration of small sets. The superior counting performance of Chinese children was thus found to be reflected in the ANS but not in the OTS.

## Introduction

Human beings are assumed to possess an evolutionarily ancient, innate system dedicated to extracting and representing approximate numerical magnitude information. This so-called approximate number system (ANS; see Piazza, 2010, for an overview) enables us to discriminate between sets of different quantities and is proposed to serve as the foundation for the acquisition of more elaborate numerical concepts (e.g., Feigenson et al., 2004). We are faster and more accurate in comparing two visually presented dot arrays with respect to their quantity the more their ratio deviates from one (e.g., van Oeffelen and Vos, 1982). The ability to discriminate between sets of different numerical quantities seems to already exist in preverbal infants (e.g., Izard et al., 2009) and undergoes a progressive refinement throughout development (Piazza, 2010; Halberda et al., 2012). Besides this developmental variation, individuals of the same age show inter-individual differences in their ability to discriminate between sets of numerical quantities. Recent meta-analyses demonstrated that these differences are linked to symbolic math performance (Chen and Li, 2014; Fazio et al., 2014; Schneider et al., 2017). According to Chen and Li (2014), this association remains significant even when considering potential moderators like general cognitive abilities, and it is comparable in strength in children and adults. On the other hand, Fazio et al. (2014) reported higher correlations for children than for adults and Schneider et al. (2017) also detected a similar but small moderating effect of age.

In addition to the ANS, a so-called object tracking system (OTS; see e.g., Piazza, 2010, for an overview) has been proposed. The OTS is assumed to enable “subitizing,” i.e., the rapid and accurate judgment of the number of small sets “at a glance,” without counting. Indeed, children can determine the number of objects in small sets of three or four items with high speed and high accuracy (Pylyshyn, 2001; Revkin et al., 2008). Similar to the ANS, the OTS undergoes a refinement throughout development and shows inter-individual differences (e.g., Reeve et al., 2012). The OTS is assumed to independently contribute to the acquisition of more elaborate numerical concepts (Feigenson et al., 2004). This is supported by studies showing an association between the ability to rapidly and accurately enumerate small sets with concurrent and future math achievement (e.g., Reeve et al., 2012; Gray and Reeve, 2014; Major et al., 2017). Dot enumeration tasks are typically used to assess the OTS. In these tasks, different sets of dots are presented (e.g., 1–9 dots) and the participants are asked to verbally state as quickly and as correctly as possible the respective number of dots. Based on a typical response pattern with a relatively flat slope for small sets of dots (1–3/4) and a steeper slope for larger sets of dots (4/5–9), it is assumed that at least two distinct systems are involved: a subitizing system (OTS) and a counting system (see e.g., Major et al., 2017). According to Piazza (2010), the number of objects in sets with more than three or four items can indeed only be assessed using exact counting or approximate estimation.

Cross-national assessments of mathematical achievement have repeatedly demonstrated that Chinese children outperform their non-Chinese peers at various ages (e.g., Wang and Lin, 2009, 2013; Mullis et al., 2012; OECD, 2013). This superior Chinese performance has been attributed to different factors including number naming systems, cultural beliefs and values, parental involvement, as well as educational systems and practices (Ng and Rao, 2010). Cross-national differences seem to emerge even before children enter elementary school. A study by Miller et al. (1995), for example, revealed that 4-year-old Chinese children can count much higher than their American peers. Moreover, Aunio et al. (2008) compared Chinese, English, and Finish preschool children’s performance in the Early Numeracy Test (ENT; Van Luit et al., 1994). According to the authors, the ENT assesses children’s use and understanding of numbers (so-called counting skills) as well as children’s understanding of quantities and relations (so-called relational skills). Counting skills were assessed by probing children’s knowledge of cardinal and ordinal numbers up to 20 (e.g., “Count on from 9 to 15”). Relational skills were assessed by asking children to compare two non-equivalent cardinal or ordinal situations from given pictures (e.g., “Here you see Indians. Point out the Indian who has less feathers than this Indian with bow and arrow”). Chinese children showed better counting skills and better relational skills than their non-Chinese peers (Aunio et al., 2008). In a related study with 4- to 7-year-old participants, Chinese children showed better counting skills than Finnish children irrespective of age, whereas only older Chinese children outperformed their Finnish counterparts in relational skills (Aunio et al., 2006). In sum, there exists ample evidence that Chinese children have more elaborated numerical concepts than their non-Chinese peers. Whether similar cross-national differences exist with regard to the ANS and the OTS, however, remains an open question.

To the best of our knowledge, there is only one study investigating differences in the ANS between Chinese and non-Chinese preschool children. Rodic et al. (2014) compared 5- to 7-year-old children from China, Kyrgyzstan, Russia, and the UK. They assessed simple arithmetic skills, the ANS, and other skills assumed to be related to the development of arithmetic skills, i.e., number naming, symbolic numerical magnitude comparison, and dot enumeration. The dot enumeration task evaluated children’s ability to map a number of dots to Arabic numerals and therefore did not directly assess the OTS. While the Chinese children significantly outperformed all other groups in the arithmetic tasks, this result was not (exactly) mirrored in the non-symbolic numerical magnitude comparison task (assessing the ANS). While Chinese children showed better non-symbolic numerical magnitude comparison performance than UK, Dungan, and Kyrgyz children, they did not significantly outperform Russian children. According to Rodic et al. (2014), the observed small advantage of Chinese and Russian children in the non-symbolic numerical magnitude comparison task supports the view that the link between the ANS and mathematical skills is relatively weak and potentially reversed (mathematical skills affecting the ANS). Meta-analytic findings by Chen and Li (2014) provide evidence for both directions of influence: non-symbolic numerical magnitude processing skills predict later math performance (*r* = 0.24, based on six longitudinal samples), but they can also be predicted by earlier math performance (*r* = 0.17, based on five longitudinal samples).

While the abovementioned findings show that Chinese children have better arithmetic skills and more elaborate numerical concepts than their non-Chinese peers, they do not deliver any clear evidence as to whether the proposed underlying systems, namely the ANS and the OTS, are more elaborate in Chinese children than in their non-Chinese peers. In the present study, we investigated this question by comparing Chinese and German preschool children with regard to their performance in a non-symbolic numerical magnitude comparison task (assessing the ANS) and in an enumeration task (assessing the OTS). In addition, we compared children’s counting skills. To assure that possible between-group differences could not be ascribed to differences in more general performance factors, we also assessed reasoning abilities and processing speed. We did not assume that Chinese and German children differ in their ANS/OTS independently of their learning experience. Based on the assumption that mathematical learning affects children’s ANS (see Rodic et al., 2014), we hypothesized that Chinese children not only have better counting skills than their German peers but also have better non-symbolic numerical magnitude processing skills. With regard to the OTS, we did not expect any difference between Chinese and German children, as we were not aware of evidence for an influence of mathematical learning experiences on the OTS.

## Materials and Methods

### Participants

The German sample consisted of 37 children (20 females, mean age 60 months, range 49–74 months) recruited from different kindergartens in the region of Frankfurt am Main. The Chinese sample consisted of 37 children (18 females, mean age 59 months, range 48–70 months) recruited from different kindergartens in the region of Beijing. Written and informed consent was obtained from the parents of all participating children. Children additionally provided verbal assent to participate in the study and were compensated for participation (e.g., by receiving a pencil). Our study was not approved by an ethics committee. This is due to the fact that data acquisition for our study started at a time when it was not common practice to apply for an ethics committee approval for psychological studies involving only cognitive measures like ours.

### Procedure

All participants were tested individually and performed the tasks in the following order: non-symbolic numerical magnitude comparison, enumeration, processing speed, counting, and reasoning. Computerized tasks (non-symbolic numerical magnitude comparison, enumeration, and processing speed) were programmed and controlled using Presentation^®^ software (Neurobehavioral Systems, Inc.)

### Non-symbolic Numerical Magnitude Comparison Task

Sets of black dots were presented in two white squares on the left- and the right-hand sides of the screen. On each trial, one of the white squares contained 32 dots (reference numerosities) and the other one 14, 20, 26, 38, 44, or 50 dots (deviants). This resulted in six different comparison pairs. Each of the six comparison pairs appeared eight times, four times with the reference numerosity on the left and four times on the right-hand side. Every single comparison pair had a unique configuration of dots. The dot sets were created using a Matlab script by Gebuis and Reynvoet (2011) which varied different visual properties of the stimuli [i.e., area extended (convex hull), total surface (the aggregate surface of all dots in one array), density (area extended/total surface), item size (average diameter of the dots presented in one array), and total circumference (circumference of all dots in one array, taken together)] so that no single visual cue was informative about numerical magnitude across all trials. Each of the five different visual cue conditions involved trials in which the respective visual cue was congruent or incongruent with the numerical dimension. Children were asked to indicate, without using counting strategies, the side of the larger numerical magnitude by pressing the left CTRL-button of the computer keyboard with their left index finger when it was larger on the left-hand side and by pressing the right CTRL-button using their right index finger when it was larger on the right-hand side. Reaction times (RT) and errors (ER) were recorded, and the instruction stressed both speed and accuracy. The order of trials was pseudo-randomized to avoid consecutive identical comparison pairs. The experiment started with six warm-up trials (stimuli: 50 vs. 32, 32 vs. 14, 26 vs. 32, 38 vs. 32, 32 vs. 44, 20 vs. 32; no feedback, data not recorded), followed by 48 experimental trials (6 comparison pairs × 8 repetitions). The experimenter pressed a button to start a trial, whereupon a black screen was presented for 1,000 ms. After the black screen had vanished, the target appeared until a response was given, but only up to a maximum duration of 6,000 ms. If no response was given, a trial was classified as erroneous. No feedback was given regarding the correctness of responses. Mean RT and mean ER were used as individual markers of the ANS (see, e.g., Inglis and Gilmore, 2014, for a discussion on different indices of the ANS). Correct responses were used for computing mean RT. Response times below 200 ms were excluded from further analysis. This trimming resulted in 0.06% of response exclusions for Chinese participants and in 0.28% of response exclusions for German participants.

### Enumeration

Sets of dots were presented in a white square in the center of the screen. On each trial, the white square contained 1, 2, 3, 4, 5, 6, 7, 8, or 9 dots. Each number of dots appeared two times and every single stimulus had a unique configuration of dots. Children were asked to verbally state as quickly and as correctly as possible the respective number of dots. To assess RT, the examiner pressed a button on an external device as soon as the child began to verbalize the answer. Then, a black screen appeared, while the examiner recorded the answer given by the child. Afterward, a new stimulus was presented. Targets appeared until the child gave an answer. No feedback was provided regarding the correctness of responses. The experiment started with four warm-up trials (stimuli: 4, 2, 8, 5; no feedback, data not recorded), followed by 18 experimental trials in total. The order of trials was pseudo-randomized so that the number of dots was not identical on consecutive trials. Mean RT and ER as well as RT slopes for sets of dots in the subitizing range were used as individual markers of the OTS. Correct responses were used for computing mean RT. ER in the subitizing range can be assumed to be very low, but from our point of view, it is still important to consider ER in the subitizing range, since it cannot be excluded from the outset that there are no group differences in this respect. In addition, mean ER as well as ER slopes for the enumeration of sets of dots beyond the subitizing range were analyzed.

### Counting

Children were asked to recite the number word sequence from 1 to 30. The last number that was counted correctly was used to estimate children’s counting skills.

### Reasoning

Raven’s Colored Progressive Matrices (CPM; Bulheller and Häcker, 2002) were used to assess inductive reasoning. The CPM is an untimed power test consisting of 36 colored diagrammatic puzzles, each with a missing part which has to be identified from a choice of six. Total scores ranging from 0 to 36 are reported for each child.

### Processing Speed

A visual detection task was used to assess individual processing speed. Children were instructed to press the space bar of the computer’s keyboard as soon as possible whenever an “X” appeared in the center of the screen. The target appeared until a response was given, but only up to a maximum duration of 3,000 ms. The task comprised 10 experimental trials with varying inter-trial intervals (2,000, 3,500, 5,000, 6,500, or 8,000 ms). Correct responses were used for computing mean RT. If no response was given, a trial was classified as erroneous. Mean ER in the visual detection task was low (Chinese children: 0.0%; German children: 0.5%) and not further analyzed.

### Analyses

To assess the effect of ratio between the two to-be-compared numerical magnitudes in the non-symbolic numerical magnitude comparison task, we collapsed trials with deviants smaller than the reference (14, 20, 26) and trials with deviants larger than the reference (38, 44, 50) into three levels of ratio [14/50 vs. 32 (ratios = 0.4375/1.5625), 20/44 vs. 32 (ratios = 0.625/1.375), and 26/38 vs. 32 (ratios = 0.8125/1.1875)] and used polynomial linear trend analyses for collapsed ratios separately for ER and RT. Moreover, we used two-sample *t*-tests to assess differences between Chinese and German children with regard to age, reasoning, processing speed, counting skills, mean RT/ER in the non-symbolic numerical magnitude comparison task, as well as mean RT/ER and RT slopes for the enumeration of sets of dots in the subitizing range. In subsequent analyses, we used two-sample *t*-tests to compare Chinese and German children with regard to mean ER and ER slopes for the enumeration of sets of dots beyond the subitizing range. The raw data supporting the conclusions of this manuscript will be made available by the authors, without undue reservation, to any qualified researcher.

## Results

Demonstrating the signature of the ANS, ER in the non-symbolic numerical magnitude comparison task decreased the more the ratio between the two to-be-compared numerosities deviated from one [Chinese children: 26/38 vs. 32: ER = 45%, 20/44 vs. 32: ER = 31%, 14/50 vs. 32: ER = 23%; *F*(1, 36) = 72.85, *p* < 0.001, $\eta_{p}^{2}$ = 0.67; German children: 26/38 vs. 32: ER = 47%, 20/44 vs. 32: ER = 36%, 14/50 vs. 32: ER = 29%; *F*(1, 36) = 75.75, *p* < 0.001, $\eta_{p}^{2}$ = 0.68]. On the basis of RT in the non-symbolic numerical magnitude task, a significant linear trend was found for German children [26/38 vs. 32: RT = 1,394 ms, 20/44 vs. 32: RT = 1,641 ms, 14/50 vs. 32: RT = 1,503 ms; *F*(1, 36) = 10.48, *p* < 0.01, $\eta_{p}^{2}$ = 0.23] but not for Chinese children [26/38 vs. 32: RT = 1,327 ms, 20/44 vs. 32: RT = 1,422 ms, 14/50 vs. 32: RT = 1,371 ms; *F*(1, 36) = 0.79, *p* = 0.379, $\eta_{p}^{2}$ = 0.02]. German children unexpectedly showed fastest RT when the ratio between the two to-be-compared numerosities was least different from one (26 or 38 vs. 32).^1^ There was, however, no indication of a speed-accuracy trade-off in German children (*r* = 0.26, *p* = 0.123).

**Figure 1:**
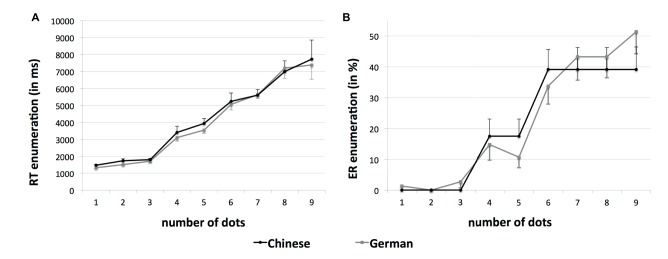
Reaction times (RT) for correct responses and error rates (ER) in the enumeration task. **(A)** RT (in ms) separately for Chinese and German children as a function of the number of dots. The sample size varies depending on the condition (number of dots), because some children did not respond correctly in all trials of a specific condition and thus RT for correct responses could not be determined. The sample size of Chinese and German children of the different conditions is as follows: number of dots = 1, 37 Chinese and 37 German children; number of dots = 2, 37 Chinese and 37 German children; number of dots = 3, 37 Chinese and 37 German children; number of dots = 4, 33 Chinese and 34 German children; number of dots = 5, 33 Chinese and 37 German children; number of dots = 6, 29 Chinese and 32 German children; number of dots = 7, 27 Chinese and 24 German children; number of dots = 8, 27 Chinese and 27 German children; and number of dots = 9, 26 Chinese and 23 German children. **(B)** ER (in %) separately for Chinese and German children as a function of the number of dots. Error bars depict one standard error of the mean.

In the enumeration task, some children did not respond correctly in all trials of a specific condition and thus RT for correct responses could not be determined for all participants in each condition (see Figure 1). When considering RT for correct responses as well as ER in the enumeration task, both Chinese and German children showed a typical response pattern (see Figure 1), with a relatively flat slope for small sets of dots (1–3) and a steeper slope for larger sets of dots (4–9). We interpreted these results as an indication for a subitizing range of 1–3 in both groups of children. Within the subitizing range, it was possible to determine RT for correct enumerations in each of the different conditions. Mean RT and ER as well as the best-fitting regression lines for each child’s RT were calculated for this range. Beyond the subitizing range (4–9), it was not possible to determine RT for correct enumerations in each of the different conditions. Accordingly, ER (in %) as well as ER slopes were calculated for this range.

While Chinese and German children did not differ significantly with regard to age [*t*(72) = 0.13, *p* = 0.897, *r* = 0.02], reasoning abilities [*t*(64) = −1.39, *p* = 0.168, *r* = −0.16], or processing speed [*t*(72) = −0.87, *p* = 0.388, *r* = −0.10], Chinese children were able to count significantly higher [*t*(72) = −3.16, *p* = 0.002, *r* = −0.34]. This superior counting performance of Chinese children was accompanied by a higher accuracy in the non-symbolic numerical magnitude comparison task [*t*(72) = 2.04, *p* = 0.046, *r* = 0.23].^2^ In contrast, RT in the non-symbolic numerical magnitude comparison task did not differ significantly between the two groups [*t*(72) = 1.49, *p* = 0.141, *r* = 0.17]. Moreover, none of the three measures used to evaluate performance in the enumeration of sets in the subitizing range showed significant group differences [mean RT: *t*(72) = −1.79, *p* = 0.077, *r* = −0.20; mean ER: *t*(36) = 1.78, *p* = 0.083, *r* = 0.20, RT slopes: *t*(72) = 0.56, *p* = 0.575, *r* = 0.07]. There was also no significant group difference regarding ER [*t*(72) = 0.12, *p* = 0.887, *r* = 0.02] and ER slopes [*t*(68) = 1.71, *p* = 0.091, *r* = 0.19] for the enumeration of sets of dots beyond the subitizing range. Table 1 displays an overview of these results. As Levene’s test indicated unequal variances for reasoning (*F* = 4.82, *p* = 0.031), mean ER for the enumeration of sets in the subitizing range (*F* = 15.28, *p* < 0.001), and ER slopes for the enumeration of sets beyond the subitizing range (*F* = 4.09, *p* = 0.047), degrees of freedom were adjusted.

**Table 1:** Comparison of Chinese and German children.

	Chinese children			German children			*p* (two-sided)
	M	SD	SE	M	SD	SE	
Age	59	7.29	1.20	60	6.99	1.20	*p* = 0.90
Reasoning	19	6.28	1.03	17	4.40	0.72	*p* = 0.17
Processing speed	689	224	37	652	144	24	*p* = 0.39
Counting	27	6.42	1.06	23	6.22	1.02	*p* < 0.01
RT comparison	1,373	424	70	1,513	380	62	*p* = 0.14
ER comparison	32.77	11.46	1.88	37.61	8.84	1.45	*p* < 0.05
RT enumeration 1–3	1,678	430	71	1,522	309	51	*p* = 0.08
ER enumeration 1–3	0	0	0	0.01	0.05	0.01	*p* = 0.08
RT slope 1–3	161	208	34	190	246	40	*p* = 0.58
ER enumeration 4–9	31.98	30.40	5.00	32.88	23.73	3.90	*p* = 0.89
ER slope 4–9	4.94	7.30	1.20	8.26	9.27	1.52	*p* = 0.09

Descriptive statistics and *p* from two-sample *t*-tests comparing Chinese and German participants with regard to age (in months); reasoning abilities; processing speed (in ms); counting skills; mean RT (in ms) and mean ER (in %) in the non-symbolic numerical magnitude comparison task; mean RT (in ms), mean ER (in %), and RT slopes for the enumeration of sets of dots in the subitizing range (1–3); and mean ER (in %) as well as ER slopes for the enumeration of sets of dots beyond the subitizing range (4–9).

*n* = 74 (37 Chinese and 37 German children).

In *post hoc* analyses, Pearson correlation coefficients were employed to examine associations between ER in the non-symbolic numerical magnitude comparison task and counting skills in both groups. No significant correlation was found for both Chinese (*r* = −0.001, *p* = 0.994) and German children (*r* = −0.30, *p* = 0.072). Using the Fisher r-to-z transformation to compare the correlation coefficients of both groups directly did not reveal a significant difference (*r* = −0.001 vs. *r* = −0.30; *p* = 0.203).

## Discussion

We compared Chinese and German preschool children regarding their performance in a counting task as well as in a non-symbolic numerical magnitude comparison task assessing their ANS and in an enumeration task assessing their OTS. Chinese children showed better performance in the counting task, which is in agreement with previous findings (e.g., Miller et al., 1995). This superior counting performance was accompanied by a better performance in the non-symbolic numerical magnitude comparison task: Chinese children were more accurate in comparing two visually presented dot arrays with respect to their quantity, while showing similarly short response times as German children. Thus, Chinese preschool children were not only able to count higher, but also showed a better performance in a task assessing the ANS. These performance differences cannot be ascribed to differences in general cognitive abilities as Chinese and German children showed similar reasoning abilities and a similar processing speed.

Group differences with regard to the OTS were statistically not significant. Although there was a trend toward fewer errors in Chinese compared to German children during the enumeration of sets of dots in the subitizing range, there was also a trend toward longer reaction times in Chinese children. Similarly, there were no significant group differences with regard to the enumeration of sets of dots beyond the subitizing range (4–9). There was, however, a trend toward a steeper error rate slope in German compared to Chinese children. This might be seen as a further indication of better counting skills of Chinese children. This interpretation must, however, be taken with caution because enumerating sets of dots beyond subitizing range may not only involve counting but also other processes like approximate estimation (Piazza, 2010). Most importantly, the findings of this study reveal that there is no clear indication of advantages for Chinese children in terms of enumerating small sets of items in the subitizing range.

In accordance with previous findings by Rodic et al. (2014), the observed advantage of Chinese children in the non-symbolic numerical magnitude comparison task is statistically significant, but the associated effect size is small (*r* = 0.23). Rodic et al. (2014) assumed a relatively small influence of the ANS on the acquisition of mathematical skills. Meta-analytic findings by Chen and Li (2014) support this view by revealing a small but significant correlation (*r* = 0.20) between the performance in non-symbolic numerical magnitude comparison tasks and mathematical skills. In the present study, no significant correlation between children’s performance in the non-symbolic numerical magnitude comparison task and their counting skills could be observed in both groups. A possible reason for this finding might be that asking children to recite the number word sequence from 1 to 30 is not comprehensive enough to be used as a measure of their early mathematical skills.

Rodic et al. (2014) additionally suggested that mathematical learning affects the ANS. In line with this view, the meta-analysis by Chen and Li (2014) revealed that while non-symbolic numerical magnitude processing skills predict later math performance (*r* = 0.24, based on six longitudinal samples), they can also be predicted by earlier math performance (*r* = 0.17, based on five longitudinal samples). Non-symbolic numerical magnitude processing skills may thus be reciprocally related to mathematical learning. Consequently, the present findings may be explained by two possible underlying mechanisms—on the one hand, more precise ANS representations may enable Chinese children to develop more elaborate counting skills than their German peers. More precise ANS representations of Chinese children might be traced back to more sophisticated visual-spatial skills (Zhou et al., 2015; see also Lonnemann et al., 2019), which have been observed as early as in preschool age and which are assumed to be a consequence of learning to read Chinese characters (see McBride-Chang et al., 2011; McBride and Wang, 2015). In this regard, it has been suggested that performance in visually presented non-symbolic numerical magnitude comparison tasks depends on the ability to integrate different visual cues (Gebuis et al., 2016). On the other hand, Chinese children’s more elaborate counting skills may result in more precise ANS representations. This assumption is corroborated by findings showing better counting skills in Chinese children than in Finnish children irrespective of age, but better performance in relational skills in Chinese children only among older children (see Aunio et al., 2006). Aunio et al. (2006) assumed that Chinese children’s relative gain in relational skills is a result of the more systematic teaching of counting skills in China. Similarly, more systematic teaching and the associated higher experience and familiarity with counting among Chinese children could have led to better non-symbolic numerical magnitude processing skills compared to German children. Longitudinal studies are needed to further explore this issue. By assessing both the development of non-symbolic numerical magnitude processing skills and the development of counting skills in Chinese and German children over a longer period of time, we would gain a better understanding of the interrelationship between these skills. Moreover, it would be possible to examine whether the direction of influence changes in the course of development and to determine to what extent the developmental trajectories are culture-specific. It can, however, not be ruled out that other factors also play a role. For example, the more regular and transparent Chinese number word system may explain Chinese children’s advantage in the counting tasks (see, e.g., Ng and Rao, 2010). If Chinese and German participants attempted to count the dots presented in the non-symbolic numerical magnitude comparison task, differences in the structure of the number naming systems may explain Chinese children’s advantage in this task. Indeed, it could be argued that children tried to count the dots in the non-symbolic numerical magnitude comparison task and that the more accurate performance of Chinese children in this task is merely due to their superior counting skills. In previous studies examining preschool children’s ANS, short presentation times were used in the non-symbolic numerical magnitude comparison task in order to prevent children from using counting strategies (see e.g., Libertus et al., 2011, 2013; Mazzocco et al., 2011). In the present study, the sets of different numerical quantities were presented up to a maximum duration of 6,000 ms. Indeed, Libertus et al. (2011, 2013) as well as Mazzocco et al. (2011) used shorter presentation times but they also used smaller set sizes (Libertus et al.: 4–15; Mazzocco et al.: 1–14) which may be more likely to trigger the use of counting strategies. Nevertheless, we cannot rule out that some of our participants attempted to count some of the stimuli. However, the instruction to not to count the dots, the number of dots (14–50), and the restricted response time (6,000 ms) should have prevented this strategy in our study. Moreover, the distribution of reaction times in our study (Chinese children: *M* = 1,373 ms, SD = 424; German children: *M* = 1,513 ms, SD = 380) indicates that the participants generally identified the side of the larger numerical magnitude without using counting strategies.

With regard to reaction times in the non-symbolic numerical magnitude comparison task, it has to be noted that we unexpectedly observed no significant effect of ratio in Chinese children and a reversed effect of ratio in German children. This indicates that reaction times in non-symbolic numerical magnitude comparison tasks cannot be considered a reliable indicator of the ANS, at least in preschool children. In this regard, it has been demonstrated that accuracy/ER-based measures are more informative about the underlying ANS acuity than RT-based measures (see Dietrich et al., 2016). In addition, recent meta-analyses revealed higher correlations between non-symbolic numerical magnitude processing skills and symbolic math performance for overall accuracy/ER compared to overall RT in a non-symbolic numerical magnitude processing task (Fazio et al., 2014; Schneider et al., 2017).

The superior counting performance of Chinese children was not accompanied by a better performance of Chinese children in enumerating small sets of items in the subitizing range. This finding does not exclude a contribution of the OTS to the acquisition of counting skills. Indeed, the OTS might be a necessary condition for the acquisition of counting skills, but it does not seem to be related to the observed difference between Chinese and German children’s counting skills. Our findings also suggest that the OTS is not affected by the development of counting skills. Due to the small sample size, the results of our study must, however, be viewed with caution. Future studies are thus needed to substantiate our findings and the aforementioned suggestions.

To conclude, results from our study revealed that differences in counting performance between Chinese and German preschool children are accompanied by differences in a non-symbolic numerical magnitude comparison task used to assess the ANS, but not by differences in an enumeration task used to evaluate the OTS. A superior counting performance of Chinese children was thus found to be reflected in the ANS but not in the OTS.

## Author Contributions

JLo, SLi, JLi, SLin, MH, and SY substantially contributed to the conception and design of the work. JLo and PZ contributed to the acquisition and analysis of data. JLo, SLi, PZ, JLi, SLin, MH, and SY substantially contributed to the interpretation of data for the work and to drafting the work and revising it critically for important intellectual content. JLo, SLi, PZ, JLi, SLin, MH, and SY agreed to be accountable for all aspects of the work in ensuring that questions related to the accuracy or integrity of any part of the work are appropriately investigated and resolved.

### Conflict of Interest Statement

The authors declare that the research was conducted in the absence of any commercial or financial relationships that could be construed as a potential conflict of interest.

## Footnotes

^1^ Similar results were found when trials with deviants smaller than the reference (26, 20, 14) and trials with deviants larger than the reference (38, 44, 50) were analyzed separately: ER decreased the more the ratio between the two to-be-compared numerosities deviated from one for deviants smaller than the reference [Chinese children: 26 vs. 32: ER = 46%, 20 vs. 32: ER = 22%, 14 vs. 32: ER = 14%; *F*(1, 36) = 76.30, *p* < 0.001, $\eta_{p}^{2}$ = 0.68; German children: 26 vs. 32: ER = 48%, 20 vs. 32: ER = 27%, 14 vs. 32: ER = 19%; *F*(1, 36) = 92.13, *p* < 0.001, $\eta_{p}^{2}$ = 0.72] as well as for deviants larger than the reference [Chinese children: 38 vs. 32: ER = 44%, 44 vs. 32: ER = 40%, 50 vs. 32: ER = 31%; *F*(1, 36) = 16.82, *p* < 0.001, $\eta_{p}^{2}$ = 0.32; German children: 38 vs. 32: ER = 47%, 44 vs. 32: ER = 46%, 50 vs. 32: ER = 40%; *F*(1, 36) = 5.97, *p* < 0.05, $\eta_{p}^{2}$ = 0.14]. On the basis of RT, a significant linear trend was found for German children in the case of deviants smaller than the reference [26 vs. 32: RT = 1,391 ms, 20 vs. 32: RT = 1,605 ms, 14 vs. 32: RT = 1,577 ms; *F*(1, 36) = 15.72, *p* < 0.001, $\eta_{p}^{2}$ = 0.23]. German children showed fastest RT when the ratio between the two to-be-compared numerosities was least different from one (26 vs. 32). No significant linear trends were found in the other conditions [Chinese children, deviants smaller than the reference: 26 vs. 32: RT = 1,402 ms, 20 vs. 32: RT = 1,402 ms, 14 vs. 32: RT = 1,422 ms; *F*(1, 36) = 0.96, *p* = 0.759, $\eta_{p}^{2}$ = 0.003; Chinese children, deviants larger than the reference: 38 vs. 32: RT = 1,253 ms, 44 vs. 32: RT = 1,442 ms, 50 vs. 32: RT = 1,320 ms; *F*(1, 36) = 1.63, *p* = 0.210, $\eta_{p}^{2}$ = 0.04; German children, deviants larger than the reference: 38 vs. 32: RT = 1,397 ms, 44 vs. 32: RT = 1,678 ms, 50 vs. 32: RT = 1,428 ms; *F*(1, 36) = 0.43, *p* = 0.518, $\eta_{p}^{2}$ = 0.01].

^2^ Performance in trials with ratios close to 1 (i.e., 26/38 vs. 32) was nearly at the chance level of 50% [Chinese children: ER = 45%, German children: ER = 47%]. In order to rule out that the reported group difference is not exclusively due to performance differences in these trials, we compared mean ER only for trials with the other ratios used (i.e., 20/44 vs. 32 and 14/50 vs. 32). In line with the results based on all trials, there was a significant group difference: Chinese children answered more accurately than German children [Chinese children: ER = 27%, German children: ER = 33%, *t*(72) = 2.11, *p* = 0.039, *r* = 0.24].
